# *Bioscience learning in nursing*: a cross-sectional survey of beginning nursing students in Norway

**DOI:** 10.1186/s12912-019-0394-3

**Published:** 2020-01-02

**Authors:** Aud Emelie Evensen, Hildfrid Vikkelsmoe Brataas, Guanglin Cui

**Affiliations:** Nord University, Faculty of Nursing and Health Sciences, Høgskolevegen 27, 7600 Levanger, Norway

**Keywords:** Student, Nursing, Learning, Motivation, Instructional methods, Quality improvement

## Abstract

**Background:**

Taking bioscience courses such as anatomy and physiology (A&P) is important for the development of nursing competence, but learning such subjects is also a challenge for many students. Nursing students’ motivation, academic performance and exposure to different teaching methods may influence the learning process.

**Methods:**

A descriptive survey was conducted with first-year nursing students at a university in Central Norway to explore their motivations, academic performance, and responses to various teaching methods used in an A&P course.

**Results:**

The study provided insight into nursing students motivation, academic performance, and responses to various teaching approaches. 57 students participated in the survey and 91 % of them passed the course. The majority (61.4%) reported that classroom lecture was the most efficient and appreciated teaching method. Independent study was significantly associated with higher A&P exam grades (*p*-value < 0.05).

**Conclusion:**

The survey suggests a need for further research about the quality, and presentation of anatomy and physiology units.

## Background

Taking bioscience courses such as anatomy and physiology (A&P) is of importance for the development of nursing competence, but understanding such complex subject matter is also a challenge for many nursing students [1]. Several factors may affect the learning process. For example, entry scores, motivation, self-efficacy, study skills, and age at entry seem to have a significant influence on the learning process [2–4]. The majority of new nursing students are 19–20 years of age, passing directly from middle school to nursing education without any work experience in the health field. Some of them may fail to realize the importance of A&P in their competence development [5, 6]. If students lack motivation for acquiring competence, they may spend little time studying the subject matter, resulting in lower grades [7, 8]. Study strategies, including the amount of time spent on independent study, may be motivated by the goal of obtaining the nursing degree, rather than acquiring nursing knowledge [8]. Learning theory points out that older students, more so than with adolescents, recognize the importance of acquiring the knowledge needed to master future tasks [9]. Consequently, a challenge for teachers is to convey the importance of A&P knowledge in the nursing profession in ways that motivate students of all ages and learning styles.

Teaching approaches may have a significant influence on the learning process [10]. In order to improve learning outcomes, teachers need to understand student perceptions about the degree of difficulty in the A&P course content. Quality improvement work in nursing education that focus on the best possible ways to support comprehension of life-science materials, should also take into account student motivations, learning strategies, and goals [11]. According to Deming’s quality development theory, data from the service recipients is essential for continuous quality development [12].

The nursing education program on the campus of a university in Central Norway has focus on quality improvement of the A&P course for first-year nursing students. The teaching approach this program is comprised of classroom lectures, group tutorials, and seminars. Classroom lectures address the most important A&P content. Small group tutorials consist of 5–6 students per group and teachers who provide help and answer questions posed by the students [13]. Seminars consist of groups with about 30 students. Some students present A&P topics and get feedback from other students and teachers. Tutorials and group seminars, designed to complement specific lectures, aim to improve student understanding of A&P topics [14]. These approaches, coupled with independent study, may help students to facilitate the learning process in order to acquire important A&P knowledge.

As a basis for evaluation and quality improvement of A&P courses, feedback information directly obtained from students, may provide insight into the learning process and perceptions of various educational approaches. The purpose of this study was to gain knowledge of students’ study motivation and the relationships between grades and and hours studying per week, and students’ experiences with various teaching methods used in the first year nursing A&P course. The long-term purpose of this study is to develop appropriate tools for continuous improvement.

Research Questions to be answered are:

- Did students motivation relate to exam performance?
- What were the students perceptions of content difficulty and efficacy of various teaching methods used in the A&P course?

## Methods

### Design

The study was a descriptive small-scale study of first-year nursing student motivation, performance, grades, and their experiences with three teaching methods used in the A&P course of the nursing education program on the campus of one Norwegian university. A questionnaire was developed specifically for these purposes.

### Participants

#### Inclusion criteria

First-year nursing students studying at one campus of a Norwegian university who had completed the A&P course in the nursing education program and taken the A&P examination. 140 students met these inclusion criteria. A practical selection method with convenience sampling was used. All first-year nursing students who met on campus on a selected day were included. 93 out of 140 students in the class met on the sampling day, and were asked to participate in the survey. 57 students answered the questionnaire. Unresponsive students did not give any reason for their nonparticipation. Two respondents failed to provide their ages, but the mean for the remainder of the 55 students was 23, ranging from 19-to-46 years. The male/female student ratio was 5/52.

### Sampling procedure

Data collection was conducted one month after the A&P exam. A researcher informed the participants about the aim of study, its procedures, and confirmed their voluntary participation in the survey. There was time allocated in the curriculum so that those who wanted to answer the questionnaire could do so without missing any of their lectures. Both answered, and unanswered, survey sheets (devoid of identifiable information) were anonymously delivered from students to a desk in the classroom and then collected by a researcher.

### Ethics approval and consent to participate

The project was approved by the research management of the university where the study was conducted. Ethics approval were deemed unnecessary according to the Norwegian national regulations, the Norwegian Centre for Research Data (https://nsd.no/nsd/english/index.html). The project did not deal with health information, and therefore an application from the Regional Committees for Regional Medical and Health Care Ethics (https://helseforskning.etikkom.no/) was not relevant. Consent to participate: A researcher informed the class about the study and related procedures and asked students for their voluntary participation. Answers to questionnaires were considered voluntary participation. There was no participant list and the results did not include any identifying information.

### The questionnaire

A questionnaire was developed for this study based on two previously-validated surveys [14; 15]. The questionnaire covered demographic information (age and sex) and 13 main questions about previous healthcare experience, motivation, level of coursework relative to expectations, attendance, frequency of assigned worksheets and readings, and on average how many hours were spent studying for this A&P course weekly. In addition, students were asked to report their final grades on the A&P examination. In order to obtain information on their experiences of classroom lectures, tutorials, and group seminars, the questionnaire was based on the Meehan-Andrews’ study about first-year health science student learning styles [14]. Using the Likert scale, questions had three–to-five answer options. The students were also asked to choose from a list of A&P content about what they considered the most-difficult content. Finally, the questionnaire provided an opportunity to provide additional comments, in either English or Norwegian. Comments were analysed using traditional content analyses, quantifying the meaning that the aggregated messages communicated [15]. The questionnaire was in English.

### Statistical analyses

Utilizing descriptive statistics, the statistical analyses were done using Prism 5.0. Frequencies, percentages, mean, and range were calculated. With a sample size of 57, no comparisons were made between gender or age groups. Given the probability level of 0.05 and an anticipated effect size (Cohen’s *d*) of between 0.7 and 0.8, the minimum required total sample size was 52 [16].

A chi-square test was used to determine if the two categorical variables independent study (‘out of school time use per week in learning A&P’) and ‘A&P exam scores’ were related. Observed study time and expected (i.e., normally distributed study time) were cross tabulated and the probability of the independence of the distribution of data was tested. Null-hypothesis was that each student’s independent study per week was independent of the student’s exam score. Chi score statistics: X^2^ score = ∑ $\frac{\left( Observed- Expected\right)2}{Expected}$ . *P* > 0.05 means that the distribution of answers based on the two variables were statistically different, while *P* < 0.05 indicated statistical coincidence.

## Results

Of the 93 students that was asked to participate, 57 responded, response rate 61.3%.

### The a&P exam grades

In the Norwegian university grade system: ‘A’ is the highest grade and ‘F’ indicates failure. For this student sample (*n* = 57), the A&P exam-grade distribution ranged from A: 5 (9%), B: 20 (35%), C: 14 (24%), D: 11 (19%), E: 2 (3%), to F: 5 (9%). Those who received exam scores of A, B, and C constituted of 58% of the sample (39 of 57 students), while 18 (31.6%) of the students achieved grades lower-than C (distributed by 5F, 2E, 11D).

### Motivation and amount of independent study used in learning a&P

The students where asked if they would continue their studies after the A&P curse, and all students were very likely or somewhat likely to continue with their study. About motivation to learn A&P, there were 34 students (59.7%) who were very motivated to succeed and 19 (33.3%) were somewhat motivated to succeed. Only 3 (5.3%) were neither motivated nor unmotivated, and 1 (1.8%) was somewhat unmotivated to do well.

Attendance at A&P lectures was reported to be high in this sample. There were 37 (64.9%) who attended the A&P class every time and 20 (35.1%) almost every time (*n* = 57). Asked” “How often do you do the assigned worksheets/readings during the A&P class period?” 50 (88%) answered “every time,” or “almost every time”; 2 (3.5%) reported “most of the time” and 5 (8.8%)” sometimes”.

Independent A&P study per week varied from < 1 h (*n* = 4) to 1~3 h (*n* = 14), to 3–6 h (*n* = 17), to 6~9 h (*n* = 8), to more than 9 h (n = 14). Using chi-square testing, the null hypothesis was that each student’s own A&P study time was independent of the person’s A&P exam score. Chi score 57.17, critical value 31.5 explored that more time spent was associated with better grades of A&P, probability calculated with degrees of freedom (5–1)(6–1) = 20, *p*-value .05. Thus, there was statistical probability of correlation between time spent studying and higher examination scores.

### Feedback about teaching approaches

Student responses to different pedagogical strategies in the teaching of A&P are listed in Table 1. One key finding from the study was that the majority of students reported that classroom lectures were the most efficient approach to teaching the material, and generally appreciated. A majority of students (61.4%) reported lectures as the most effective teaching method most of the time or all the time. In addition, 31.6% of the students felt classroom lectures were a useful learning experience “half of the time.” Only two students (3.5%) thought that lectures did not work, and two (3.5%) of the students felt that classroom lectures were of little use.

**Table 1:** Students’ experience with teaching methods used in the A&P course

Frequency (%) statement confirming*						
Statement about teaching method	NT	ST	HT	MT	AT	*P* value
*Classroom lectures*						
Classroom lectures were a useful learning experience for A&P	2 (3.5)	2 (3.5)	17 (29.8)	29 (50.9)	7 (12.3)	*< 0.01*
New ideas were introduced in the lectures	4 (7.4)	13 (24.1)	17 (31.5)	20 (37.0)	0 (0)	*< 0.01*
Tutorials						
I get feedback which helped me learn in tutorials	3 (5.3)	15 (26.3)	9 (15.8)	23 (40.4)	7 (12.2)	*< 0.01*
Tutorials allowed me to verbally demonstrate my understanding of this topic in my own words^∗^	0 (0)	11 (19.3)	13 (22.8)	24 (42.1)	9 (15.8)	*< 0.01*
Group seminar						
Group seminar allowed me to interact and socialize with other students	3 (5.3)	12 (21.1)	11 (19.3)	23 (40.4	8 (14.0)	*< 0.01*
Integration						
Tutorial sessions helped me to understand the lecture materials^∗^	1 (1.8)	11 (19.3)	18 (31.6)	22 (38.6)	5 (8.8)	*< 0.01*
Group seminar helped me to understand the lecture materials^∗^	10 (17.5)	21 (36.8)	16 (28.1)	9 (15,8)	1 (1,8)	*< 0.01*
I have learned to make connections between this subject and others	0 (0)	3 (5.3)	17 (29.8)	31 (54.4)	6 (10,5)	*< 0.01*

*NT true none of the time, *ST* true some of the time, *HT* true half of the time, *MT* true most of the time, *AT* true all of the time

Statistical significant results obtained from chi-square test.

Regarding the efficacy of tutorials and group seminars, over half of the students reported that these two methods were working “half” or “most of the time,” while one third of students felt these methods were working “none” or only “some of the time.” Analysing the integration efficacy of tutorials and group seminars in the understanding of lecture materials, most students benefited from these two teaching approaches. About these approaches, 37 (64.%) of the students answered “I have learned to make connections between this subject and others” most, or all, of the time.

### Difficulty expectations and difficult content

Regarding the awareness of A&P difficulty, 28 students (49.1%) reported the A&P course matching their expectations of difficulty and 21 students (36.8%) anticipated it to be somewhat less difficult than expected. Only one student (1.8%) expected it to be much more difficult and 7 students (12.3%) expected it to be somewhat more difficult. These data reflected that many first-year nursing students had an insufficient expectation about the difficulty level of A&P courses when they started this class. Table 2 presents the content that students found to be most difficult. The nervous system, the kidneys and urinary tract, and base-acid balance were perceived as the three most difficult topics in A&P.

**Table 2:** First-year nursing student conceptions about the most difficult topics in A&P

Which part of contents, according to the textbook, is the most difficult?	Yes*
1. The nervous system	20
2. The kidneys and urinary tract	20
3. Acid-base balance	19
4. Hormones and metabolism	14
5. Immune system	10
6. Bone, joint, and muscle	9
7. Energy Balance	5
8. Sensory organs	5
9. Cells and tissues	3
10. Temperature regulation	2
11. Reproduction	1
12. Respiration	1
13. Circulation and hemostasis	0
14. Digestion	0

*In this part of the investigation, some students responded in more than one section.

### Additional comments for a&P teaching work

This survey allowed the students to provide additional comments about the A&P course. Nineteen students added comments and all of them mentioned something that the students complained about. Twelve comments were about group seminars, in which they complained that too much time was used for seminars. Seminars also had low teacher involvement and students complained about the inefficiency of the arrangement. Four comments complained about the textbook and three about the pedagogic quality in general.

## Discussion

As a basis for improvement in an A&P course, the study provided information about student exam grades, student motivation and performance, and student perceptions of content and teaching approaches used in the A&P course. 57 students participated, the minimum required total sample size estimated to 52 reached, given the probability level 0.05 the anticipated effect size (Cohen’s *d*) 0.7, and the desired statistical power level 0.8 [16]. On the other hand, the sample was regarded as too small to do comparisons between gender or age groups.

Gathering data from a brief 15-min in-class survey paying particular attention to perceptions among students as service recipients was an efficient way of gathering data about student motivation, learning activities, and perspectives on multiple teaching methods employed in the A&P learning process [11, 12]. The majority reported that classroom lecture was the most efficient and appreciated teaching method. Independent study was significantly associated with higher A&P exam grades. Student motivation is a vital learning and achievement determinant [3, 17]. In this study, the results showed that the majority of first-year nursing students had a very high rate of positive motivation to study in the nursing programme and understood the importance of anatomy and physiology for their future career development. All (100%) of students attended this course every time or almost every time, and 50(88%) of the students did the assigned worksheets and readings every time or almost every time during the class.

### Relationships between motivation and independent study and perceptions of teaching methods

Independent study outside of the lecture hall used for individual learning activities is another factor that influences the learning process [18]. The time that students used in the study of A&P varied from > 1 h-to-9 h per week. Individual time spent studying A&P weekly was significantly with better exam results. These findings point at possible reasons for 31.6% of the students achieving grades lower than the average of ‘C’ on the final A&P exam. The findings also indicated that independent study is of importance to the learning process. This might also reflect the notion that A&P is a very difficult basic bioscience class for nursing students [1]. Data from this survey has illustrated that around half (49.1%) of the nursing students recognized that it matched their expectations for difficulty level before starting the course. However, 36.8% of students reported that they “expected it to be somewhat less difficult” and that they did not spend enough time studying on their own. This information suggests a need for action before the course starts in order to promote realistic expectations about the difficulty of the subject matter and, at the same time, motivate students for great engagement and use of sufficient time spent on the individual learning activities. Students are adult learners [9] and therefore their motivation may come from an expectation of success in acquiring knowledge that is an important component of the nursing competence [19]. Their level of motivation to learn and their anticipation of the need for A&P knowledge in the future seem to be important for teachers’ focus in future instructions. This finding is underlined by other research [4, 5, 17, 20].

### Student perceptions of content and teaching methods used in the a&P course

In order to improve nursing education in the future, it is important to understand what students consider the most difficult A&P content. The three most difficult topics were the nervous system, kidneys and the urinary tract, and base-acid balance. These findings indicate that instructors should pay particular attention when teaching these topics, and recommend that students to spend much study time on those concepts and discuss any ambiguities with their teachers and fellow students.

Classroom lectures, small-group tutorials and seminars are the three main teaching strategies in the A&P course studied, which are also used at many other institutions [14]. The survey showed that all students attended the A&P lectures and that most of them were satisfied with this form of instruction. Traditionally, classroom lectures have been viewed as an inexpensive way of presenting concepts and the relevance of human physiology and anatomy to a large group of students, and to promote interest in the subject matter [10, 21]. Our data revealed that small group tutorials were also working fine for most nursing students. In line with adult learning theory [9] the tutorials provided students with the opportunity to engage with course content at levels that had personal meaning for them. This method can support a deeper approach to learning, as students in small groups are encouraged to participate in critical discussions which can stimulate the learning process [13].

On the other hand, most of the negative responses were about the efficacy of group seminars. A total of 19 (33.3%) of the students had an additional comment about the fact that group seminars weren’t very useful, but rather wasted a lot of time. Such complaints reflect a need to reconsider the arrangement, design, and content used in group seminars in the future.

As teaching strategies, classroom lectures and small group tutorials were useful in learning A&P during this course, the efficient integration of the various teaching approaches helped students to understand the important points made during individual lectures, and also to make connections between subjects. Varying the use of teaching methods can also meet the need for both individual work with the subject matter [22] and the facilitation of collaborative learning [13]. Nevertheless, in the sample, 31.6% of the students achieved lower grades on the A&P exam than the average grade of ‘C.’ This points to the need to identify which students are struggling to acquire A&P knowledge, and take action to motivate them for more study, and to facilitate dialogue with teachers and students about the difficult topics that have been identified.

### Limitations

Provided that the sampling method did not lead to biased results, the study gave relevant information as a basis for the evaluation and improved quality development of an A&P course in a nursing education program on one campus of a Scandinavian university. Therefore, results are not generalizable to other programs. Taking the convenience sampling, method, and response rate into consideration, the study provided insight into the strengths and weaknesses of current teaching approaches used in an A&P course. Approximately one-third of the students in the selected class participated, and this points to the need for measures that can secure higher student participation in class surveys. Some variation in the results may be due to coincidences from sampling done during one randomly-selected class. This was a self-reported survey, so answers may involve biases. The questionnaire was based mainly on instruments developed by Sturges et al. [14] and Meehan-Andrews [20] and on their tests of validity and reliability. One weakness of the questionnaire was that it had only one question of motivation. In the future, the mapping tool should be further developed and validated for use in continuous quality improvement of A&P courses.

Course limitations should be taken into account. We studied only three commonly used teaching approaches. Other approaches may also prove useful, however. For example, e-learning methods [23].

## Conclusion

This survey provided insight about students with variable awareness of the difficulty of A&P course work, and variation in their study performance and exam results. The nervous system, the kidneys and urinary tract, and base-acid balance were reported as the three most difficult topics in A&P. Classroom lectures were reported as the most effective teaching method. A high rate of attendance for A&P lectures was reported and independent study varied from one-to-nine hours per week. These findings can be useful for planning quality improvement of A&P teaching methods in the nursing curriculum. There is a need for more research about effective teaching methods and how to promote realistic student expectations regarding time management involved with the study of anatomy and physiology.

## Data Availability

The datasets analyzed during the current study are available from the corresponding author on reasonable request.

## Abbreviations

A&P: Anatomy and physiology
